# Age-dependent kinetics of dentate gyrus neurogenesis in the absence of cyclin D2

**DOI:** 10.1186/1471-2202-13-46

**Published:** 2012-05-07

**Authors:** Anne Ansorg, Otto W Witte, Anja Urbach

**Affiliations:** 1 Hans Berger Department of Neurology, Jena University Hospital, Erlanger Allee 101, 07747, Jena, Germany

## Abstract

**Background:**

Adult neurogenesis continuously adds new neurons to the dentate gyrus and the olfactory bulb. It involves the proliferation and subsequent differentiation of neuronal progenitors, and is thus closely linked to the cell cycle machinery. Cell cycle progression is governed by the successive expression, activation and degradation of regulatory proteins. Among them, D-type cyclins control the exit from the G_1_ phase of the cell cycle. Cyclin D2 (cD2) has been shown to be required for the generation of new neurons in the neurogenic niches of the adult brain. It is differentially expressed during hippocampal development, and adult cD2 knock out (cD2KO) mice virtually lack neurogenesis in the dentate gyrus and olfactory bulb. In the present study we examined the dynamics of postnatal and adult neurogenesis in the dentate gyrus (DG) of cD2KO mice. Animals were injected with bromodeoxyuridine at seven time points during the first 10 months of life and brains were immunohistochemically analyzed for their potential to generate new neurons.

**Results:**

Compared to their WT litters, cD2KO mice had considerably reduced numbers of newly born granule cells during the postnatal period, with neurogenesis becoming virtually absent around postnatal day 28. This was paralleled by a reduction in granule cell numbers, in the volume of the granule cell layer as well as in apoptotic cell death. CD2KO mice did not show any of the age-related changes in neurogenesis and granule cell numbers that were seen in WT litters.

**Conclusions:**

The present study suggests that hippocampal neurogenesis becomes increasingly dependent on cD2 during early postnatal development. In cD2KO mice, hippocampal neurogenesis ceases at a time point at which the tertiary germinative matrix stops proliferating, indicating that cD2 becomes an essential requirement for ongoing neurogenesis with the transition from developmental to adult neurogenesis. Our data further support the notion that adult neurogenesis continuously adds new neurons to the hippocampal network, hence increasing cell density of the DG.

## Background

As one of the neurogenic zones in the adult mammalian brain, the hippocampal dentate gyrus (DG) generates neural progenitor-derived neurons throughout life. This process, known as adult neurogenesis, is modulated by various intrinsic and extrinsic factors ranging from neurotransmitters, growth factors, hormones, physical activity, learning, to seizures and other brain pathologies (reviewed in [1]). The newborn neurons have been shown to become functionally integrated into the pre-existing neuronal circuitry [2-4]. During the first weeks of life, newborn neurons express unique physiological characteristics thereby providing the network with enhanced functional plasticity [5], extensively reviewed in [6]. Whilst recent research suggests an involvement of newborn granule cells (DGC) in hippocampal function, the precise role of these cells still remains elusive.

Cyclin D2 belongs to a family of three highly homologous D-type cyclins (cyclins D1, 2 and 3) which are important regulators of cell cycle progression. Once activated, D-type cyclins associate with and thereby activate the cyclin-dependent kinases cdk4 and cdk6 [7,8]. These cyclin D-cdk complexes are deemed to execute critical functions during middle to late G1 phase and to be essential for the transition from G1 to S-phase [7-9]. Unlike many other cyclins that are expressed periodically during the cell cycle, D-type cyclins become synthesized in response to mitogens and their expression rapidly declines when mitogens are withdrawn [10-13]. Mitogenic signalling is also required for assembly and kinase activity of cyclin D-cdk complexes [10]. Consequently, D-type cyclins are regarded as constituting a molecular link between the extracellular environment and the cell cycle machinery.

Although different D-type cyclins can be detected in a particular cell type, they exhibit distinct cell- and tissue-specific expression patterns both during development and in adulthood [13-15]. Studies from knock out mice with deletions of one, two, or all G1 cyclins revealed remarkably normal morphogenesis at least until midgestation (reviewed in [16]), indicating a considerable degree of functional redundancy and compensatory capacity [17-23]. Mice lacking just a single D-type cyclin are viable, exhibiting only narrow, tissue-specific defects. Severe phenotypic abnormalities are observed only in those tissues expressing just one D-type cyclin, which feature no ability to compensate, i.e. by upregulating an alternative D-cyclin [19,22-25].

In the present work we analyzed postnatal and adult hippocampal neurogenesis in mice lacking cD2 (cD2KO). These animals have been reported to exhibit female sterility, hypoplastic testes in males [22], as well as cerebellar abnormalities [24] and impaired proliferation of B-lymphocytes [26]. Importantly, Kowalczyk and coworkers [27] revealed a requirement of cD2 for adult neurogenesis. They showed that proliferation is impaired in the neurogenic zones of adult cD2KO mice whilst developmental neurogenesis at postnatal day 5 appeared to be close-to normal. The aim of our study was to determine the kinetics of postnatal and adult neurogenesis as well as the precise age at which neurogenesis ceases in the absence of functional cD2. We characterized cD2KO and WT mice at seven time points during the first 10 months of life, and determined their potential to generate new neurons in the DG.

## Results

**Figure 1:**
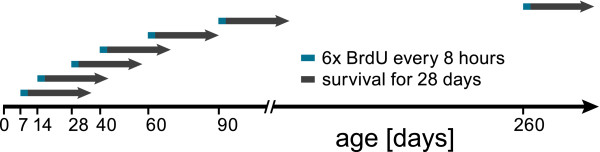
**Experimental scheme.** Starting at the ages indicated, different groups of mice received repeated BrdU-injections and were allowed to survive for 28 days.

In the present study we examined cell proliferation, neurogenesis and morphometric parameters of the hippocampus of cD2KO and WT litters at seven time points during the first 10 months of life (Figure 1).

### Morphometry

**Figure 2:**
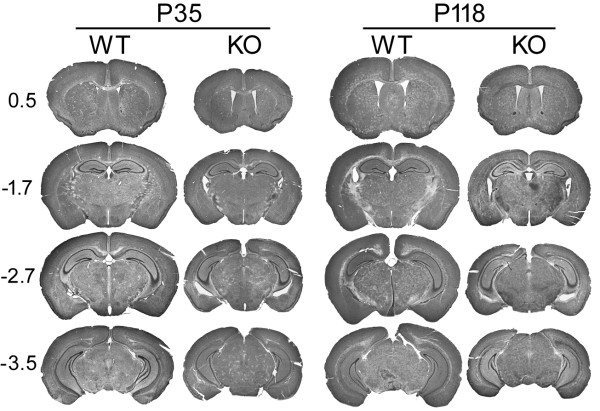
**Examples of Nissl-stained sections spanning the rostro-caudal axis of the brain illustrating differences in overall brain structure of WT and cD2KO mice.** Positions relative to bregma are marked on the left. The gross morphology of cD2KO brains appears to be close to normal but brains of cD2KO mice are smaller than that of their WT litters. Size differences are already apparent at P35.

**Figure 3:**
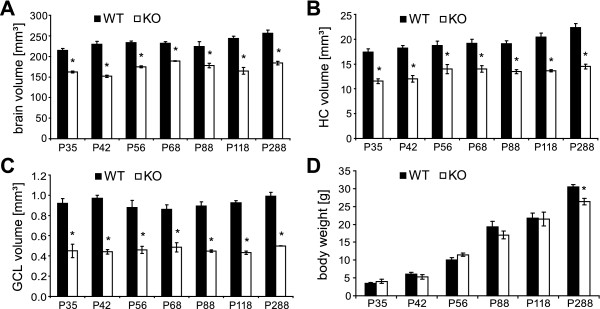
**Differences in brain structure and body weight of WT and cD2KO mice.** Estimations of the total (bilateral) volume of brain **(A)**, hippocampus **(B)** and the dentate GCL **(C)** reveal substantial reduction of these structures due to the lack of functional cD2 (*p < 0.001). Differences are already apparent at P35. **(D)** The body weight of cD2KO mice is similar to that of WT litters, except at an age of P288 (*p < 0.01). Statistical significance is only marked for genotype-specific differences.

Volumetric estimation of the entire brain, hippocampus and the dentate GCL in cD2KO mice revealed significant differences compared to WT litters at all ages examined. On average, the brain was smaller by ~26% (Figures 2 and 3A), the hippocampus by ~31% (Figure 3B) and the dentate GCL by ~49% (Figure 3C). These differences were already present in 1 month-old animals (P7 group). In both genotypes, brain volume remained fairly constant over time (Figure 3A). We only detected a slight increase from P35 to P118 (*p* = 0.011) and P88 to P288 (*p* = 0.018) in WT mice, and from P42 to P68 (*p* = 0.007) and P42 to P288 (*p* = 0.029) in cD2KO mice. The volume of the HC increased continuously in WT mice, especially when comparing ages of P88 and younger to P288 (P88 vs. P288: *p* = 0.042; Figure 3B). In contrast, the HC volume of cD2KO mice showed no significant age-related differences. Similarly, the dentate GCL volume did not change with increasing age, independent of genotype (Figure 3C).

Although cD2KO mice tended to have lower body weights than WT litters, the only significant difference was observed in mutant mice at P288 with about 14% less body weight (*p* = 0.018; Figure 3D).

### Absolute number of dentate granule cells

**Figure 4:**
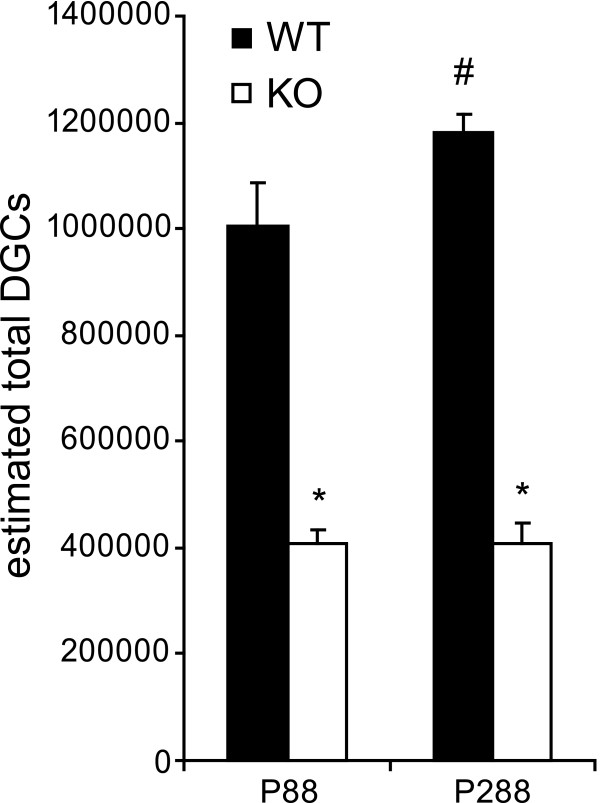
**Adult cD2KO mice have reduced numbers of dentate granule cells (DGCs).** Absolute numbers of DGCs were estimated in mice aged P88 or P288. At both ages, they were significantly reduced due to the lack of functional cD2 (*p < 0.001). In WT animals, the number of DGCs increased with advancing age (^#^*p* < 0.05). We found no evidence for such an age-related change in cD2KO mice.

The total number of DGCs in adult animals differed significantly between WT and cD2KO mice (Figure 4). At P88, cD2KO mice had ~60% fewer DGCs than their WT litters (WT: 1006057 ± 79843, cD2KO: 406455 ± 28201; p < 0.001). At P288, the number of DGCs in cD2KO mice was ~65% lower as compared to WT mice (WT: 1179307 ± 36738, cD2KO: 409150 ± 35489; p < 0.001). Moreover, the number of DGCs in WT, but not in cD2KO mice, increased between P88 and P288 (p = 0.032). For all animals the CE fell below 0.05.

### Number of BrdU-positive cells

**Figure 5:**
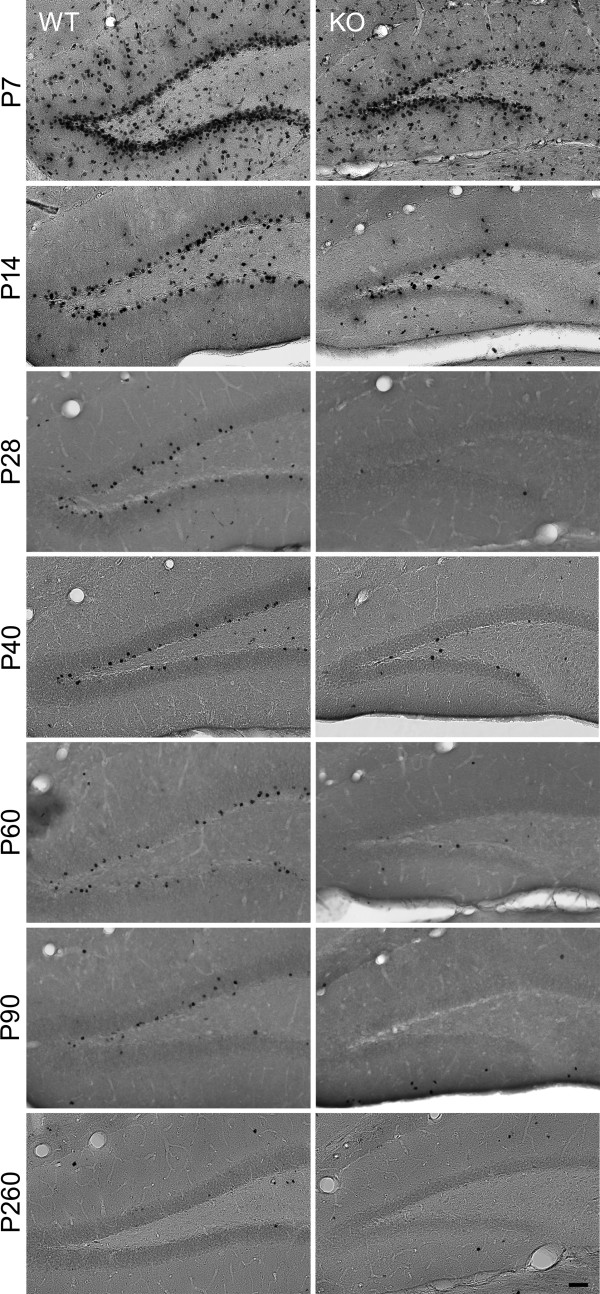
**Representative images of BrdU-immunolabeled coronal sections through the DG of cD2KO and WT mice.** BrdU-positive nuclei appear almost exclusively in the SGZ and GCL of mice that were BrdU-injected at P14 or later. When BrdU was injected at P7, many BrdU-positive cells were furthermore found scattered through the hilus and molecular layer of the DG. All slices represent approximately the same position in the rostrocaudal extension of the HC, the age of first BrdU injection is indicated on the left. Scale bar: 50 μm.

**Figure 6:**
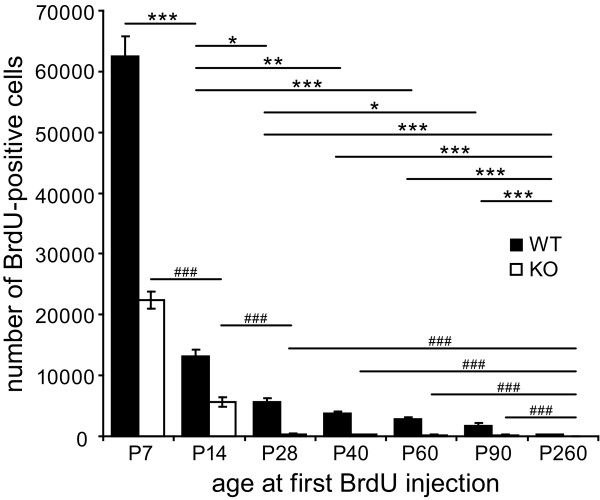
**BrdU-positive cell numbers are significantly reduced in cD2KO mice.** As compared to WT litters BrdU incorporation is significantly reduced in cD2KO mice at all postnatal ages analyzed (*p* < 0.001). CD2KO animals aged P7 at first BrdU injection have already ~60% reduced BrdU-positive cell numbers as compared to WT litters. As early as P28, newborn cells are almost completely absent in cD2KO mice. In both genotypes, BrdU-positive cell numbers continuously decline during adolescence and adulthood. Statistical differences are only indicated for age-dependent changes within WT (*) and cD2KO (^#^) groups (*p < 0.05, **p < 0.01, *** or ^###^*p* < 0.001; statistics on *ln*-transformed values).

BrdU was injected in WT and cD2KO mice of different ages (6 times at 8-hour intervals starting either at postnatal day (P)7, P14, P28, P40, P60, P90 or P260). The brains of these animals were examined 28 days later (Figure 1). BrdU-positive cell numbers were significantly reduced in the DG of cD2KO mice (*p* < 0.001), an effect that could be observed at all ages analyzed in this study (Figures 5 and 6). The difference was lowest at early postnatal ages with about 60% less BrdU-positive cells in cD2KO mice compared to WT litters (P7 and P14, *p* < 0.002). As early as in the P28 group the difference between cD2KO and WT mice reached > 93% (*p* < 0.001), with very scarce BrdU-positive cells in the DG of cD2KO animals. In both genotypes, age significantly affected BrdU-positive cell numbers, with changes fitting best to a power function (WT: *f*(x) = 668654x^-1.3727^, R^2^ = 0.9794; cD2KO: *f*(x) = 461772x^-1.8231^, R^2^ = 0.9288). BrdU-incorporation was highest in the P7 brain and subsequently declined with increasing age. The dynamic of the age-related decline in BrdU-positive cell numbers was slightly different in cD2KO and WT mice. In cD2KO mice, newborn cell numbers decreased by ~75% between P7 and P14 (*p* < 0.001) and further between P14 and P28 (~93%, *p* < 0.001). As early as at P28, BrdU-positive cells were virtually absent in these animals, with their numbers remaining roughly stable until P90, followed by a further decline towards P260 (~87%, *p* < 0.001). In contrast, WT mice started with a much higher level of cell birth (P7) and newborn cell numbers declined continuously during adulthood (Figure 6). Between P7 and P14, newborn cell numbers declined at a similar rate than in cD2KO (~79%, *p* < 0.001), while the subsequent decrease was less pronounced (~72% between P14 and P40, *p* = 0.001). BrdU-positive cell numbers continued to decline in WT by ~68% between P28 and P90 (*p* = 0.002), and by ~83% between P90 and P260 (*p* < 0.001).

Independent of genotype and age, BrdU-positive nuclei appeared preferentially in the subgranular layer (SGZ) and inner GCL (Figure 5). However, on BrdU being injected at ages ≤ P14, BrdU-positive cells also appeared scattered throughout the hilus and, in particular in the P7 group, in the molecular layer and other parts of the developing HC (Figure 5).

### Phenotype of BrdU-positive cells

**Figure 7:**
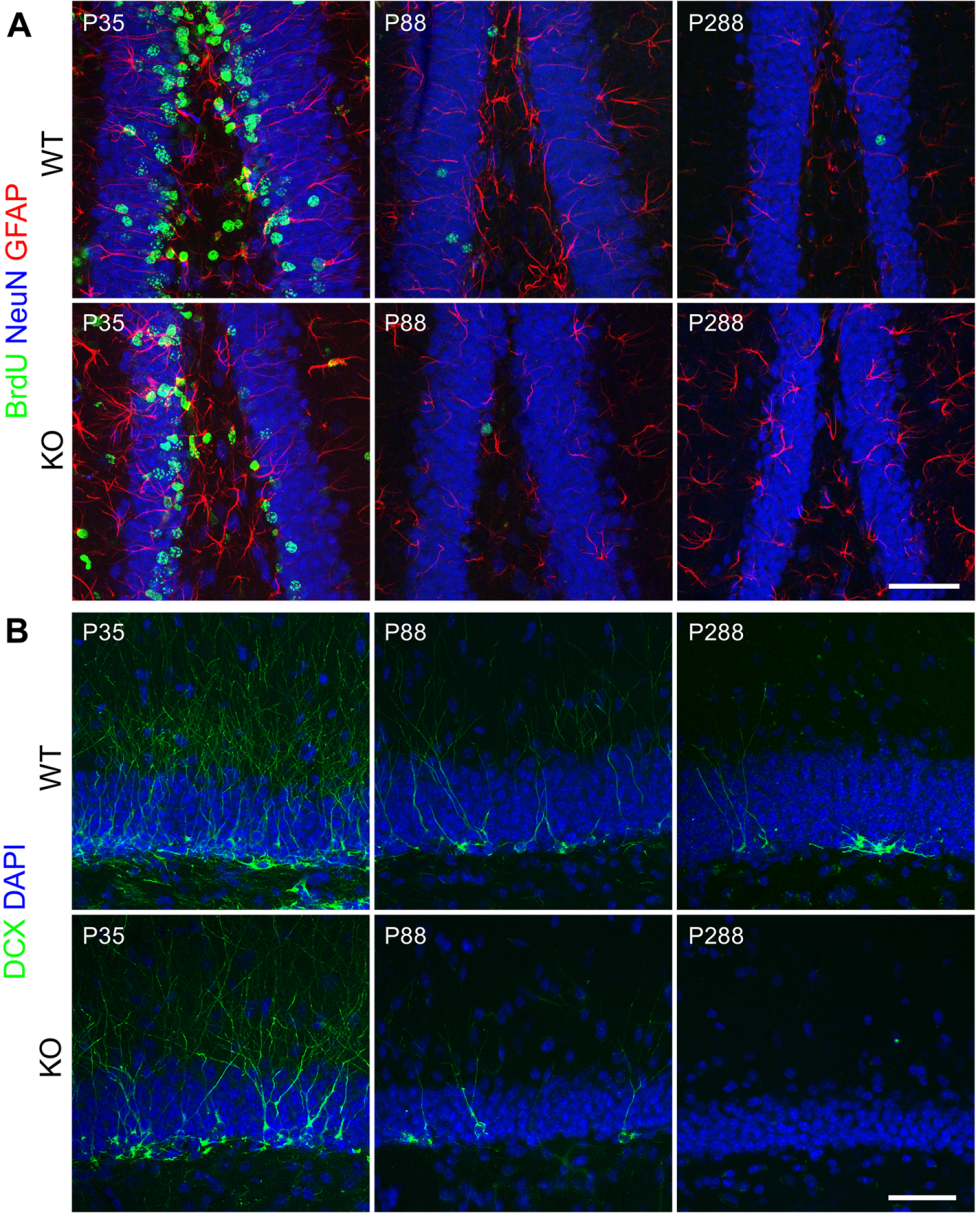
**Illustration of neurogenesis in the dentate gyrus of cD2KO and WT litters.(A)** Sections of brains aged P35, P88 or P288 were stained against BrdU (green), NeuN (blue) and GFAP (red). Irrespective of genotype, the majority of BrdU-positive cells co-labeled with NeuN indicating that neurogenesis takes place also in the DG of cD2KO mice, albeit at a much lower rate. **(B)** Representative DCX-labeling (green) at P35, P88 or P288. CD2KO mice appear to have less DCX-positive cells than WT litters. Sections were counterstained with DAPI. The images in (A) and (B) are merges of multiple confocal planes (for NeuN, GFAP, BrdU: 4–5 planes spanning a z-dimension of approximately 4.8 to 6.4 μm; for DCX: 3–4 planes spanning a z-dimension of approximately 3.6 to 4.8 μm). Scale bars: 50 μm.

To determine the potential of DG progenitor cells to differentiate into neurons we stained coronal sections against BrdU, GFAP and NeuN so as to distinguish astrocytes and putative stem cells (both expressing GFAP) from neurons (expressing NeuN; Figure 7A). Independent of genotype and age of the animals, BrdU-labeled progenitors preferentially differentiated into neurons within 4 weeks (on average 62% in WT and 57% in cD2KO; Table 1), indicating that neuronal differentiation is not affected by the lack of functional cD2. Only a small percentage (on average 5% in both genotypes; Table 1) of newborn cells expressed GFAP leaving about 35% of BrdU-positive cells with an unidentifiable phenotype (not co-localized to either NeuN or GFAP). However, when extrapolated to absolute numbers, the lack of functional cD2 resulted in a significant reduction of the number of adult-born dentate granule neurons. Genotype and age-related differences in absolute numbers of newborn neurons were similar to those observed in BrdU-positive cell counts (Table 1). For example, in the cD2KO group we found 261 new neurons that were born at P28/P29, which was ~94% less than in corresponding WT litters.

**Table 1:** Phenotype of newborn cells 28 days after BrdU delivery

		**BrdU**						**NeuN**						**GFAP**					
		**WT**			**KO**			**WT**			**KO**			**WT**			**KO**		
		mean	±	SEM	mean	±	SEM	mean	±	SEM	mean	±	SEM	mean	±	SEM	mean	±	SEM
**P7**		(n = 8)			(n = 3)														
	BrdU abs.	62466.0	±	3239.3	22394.0	±	1455.3***	---	±	---	---	±	---		---	±	---	---	±	---
	% of BrdU+	---	±	---	---	±	---	57.4	±	2.0	60.7	±	0.3	6.3	±	1.9	4.9	±	1.2	
	abs. co-lab.	---	±	---	---	±	---	35621.2	±	1643.7	13593.5	±	845.7**	3831.5	±	1060.3	1140.2	±	336.9**	
**P14**		(n = 5)			(n = 4)															
	BrdU abs.	13147.2	±	1060.1	5671.5	±	798.5**	---	±	---	---	±	---	---	±	---	---	±	---	
	% of BrdU+	---	±	---	---	±	---	64.7	±	2.2	51.0	±	5.2	1.9	±	1.2	1.2	±	0.8	
	abs. co-lab.	---	±	---	---	±	---	8480.3	±	649.6	2812.2	±	271.3**	223.0	±	141.4	84.3	±	63.7	
**P28**		(n = 4)			(n = 3)															
	BrdU abs.	5578.5	±	708.4	390.0	±	104.3***	---	±	---	---	±	---	---	±	---	---	±	---	
	% of BrdU+	---	±	---	---	±	---	73.1	±	3.8	64.8	±	2.6	0.9	±	0.1	2.6	±	2.6	
	abs. co-lab.	---	±	---	---	±	---	4100.7	±	583.4	261.1	±	88.5***	45.8	±	6.6	6.0	±	6.0	
**P40**		(n = 3)			(n = 3)															
	BrdU abs.	3726.0	±	305.2	306.0	±	51.3***	---	±	---	---	±	---	---	±	---	---	±	---	
	% of BrdU+	---	±	---	---	±	---	64.6	±	4.0	60.2	±	7.1	8.3	±	4.5	12.2	±	6.5	
	abs. co-lab.	---	±	---	---	±	---	2420.3	±	299.7	190.7	±	53.1***	313.5	±	186.6	31.3	±	17.0	
**P60**		(n = 4)			(n = 4)															
	BrdU abs.	2893.5	±	205.5	222.0	±	52.7***	---	±	---	---	±	---	---	±	---	---	±	---	
	% of BrdU+	---	±	---	---	±	---	60.1	±	4.1	46.3	±	5.7	3.8	±	2.8	0.0	±	0.0	
	abs. co-lab.	---	±	---	---	±	---	1756.7	±	219.8	101.3	±	23.6***	94.9	±	68.6	0.0	±	0.0	
**P90**		(n = 4)			(n = 4)															
	BrdU abs.	1770.0	±	354.2	205.5	±	81.5***	---	±	---	---	±	---	---	±	---	---	±	---	
	% of BrdU+	---	±	---	---	±	---	66.6	±	6.6	72.5	±	14.8	5.7	±	1.5	10.8	±	7.9	
	abs. co-lab.	---	±	---	---	±	---	1136.2	±	198.5	157.9	±	79.2***	107.0	±	30.1	29.7	±	18.4	
**P260**		(n = 4)			(n = 3)															
	BrdU abs.	300.0	±	60.1	26.0	±	6.2***	---	±	---	---	±	---	---	±	---	---	±	---	
	% of BrdU+	---	±	---	---	±	---	49.7	±	2.0	44.4	±	29.4	9.1	±	3.2	5.6	±	5.6	
	abs. co-lab.	---	±	---	---	±	---	150.0	±	33.5	13.3	±	11.4***	30.1	±	15.4	0.7	±	0.7	

Two-way ANOVA on *ln*-transformed data (*p < 0.05, **p < 0.01, ***p < 0.001 cD2KO compared to WT); Abbreviations: abs. – absolute, abs. co-lab. – absolute number of cells co-labeled with BrdU.

### Proliferating (Ki67-positive) cells and co-labeling with DCX

To evaluate the number of cells with ongoing proliferation, we stained brain sections of WT and cD2KO mice killed at P35, P88, or P288 (P7, P60, and P260 group, respectively) against Ki67, a nuclear antigen that is expressed during the G_1_, S, M and G_2_ phases of cell cycle. Ki67-positive cells were located as clusters predominantly in the SGZ, irrespective of age or genotype. Quantification revealed that the number of proliferating cells was considerably reduced in cD2KO mice (*p* < 0.001; Table 2). Additionally, Ki67-positive cell numbers were highest in the adolescent DG and declined significantly with age in both genotypes (*p* < 0.05; Table 2).

**Table 2:** Number of Ki67-positive cells and co-labeling with DCX

		**Ki67**						**DCX**					
		**WT**			**KO**			**WT**			**KO**		
		mean	±	SEM	mean	±	SEM	mean	±	SEM	mean	±	SEM
**P35**	Ki67 abs.	8247.4	±	746.1	800.0	±	82.3***	---	±	---	---	±	---
	% of Ki67+	---	±	---	---	±	---	24.1	±	2.3	9.2	±	4.6
	abs. co-lab.	---	±	---	---	±	---	1700.7	±	92.6	81.2	±	40.8***
**P88**	Ki67 abs.	4029.0	±	159.0	147.0	±	21.7***	---	±	---	---	±	---
	% of Ki67+	---	±	---	---	±	---	27.5	±	2.8	8.3	±	8.3*
	abs. co-lab.	---	±	---	---	±	---	1072.5	±	209.5	20.6	±	20.6***
**P288**	Ki67 abs.	1038.0	±	260.8	72.0	±	3.5***	---	±	---	---	±	---
	% of Ki67+	---	±	---	---	±	---	23.8	±	6.3	0	±	0**
	abs. co-lab.	---	±	---	---	±	---	365.6	±	35.6	0	±	0*

Two-way ANOVA followed by Holm-Sidak post hoc test (*p < 0.05, **p < 0.01, ***p < 0.001; cD2KO compared to WT); Abbreviations: abs. – absolute, abs. co-lab. – absolute number of cells co-labeled with Ki67.

To analyze the fraction of proliferating cells that are already determined to the neuronal lineage, we performed co-labeling against Ki67 and doublecortin (DCX), which serves as marker of putative neuronal progenitors and immature neurons. The fraction and absolute numbers of Ki67-positive cells that co-expressed DCX was considerably reduced in cD2KO mice (Table 2). In WT animals, ~25% of proliferating Ki67-positive cells were DCX-positive, irrespective of the age of the animals, whilst in cD2KO mice, ~9% of Ki67-positive cells co-expressed DCX (Table 2). At P288, we detected only sparse proliferating cells in cD2KO mice; cells clearly immunoreactive for DCX were virtually absent (Table 2, Figure 7B). In general, cD2KO mice appeared to have less DCX-positive cells than WT litters (Figure 7B).

### Number of TUNEL-positive nuclei

**Figure 8:**
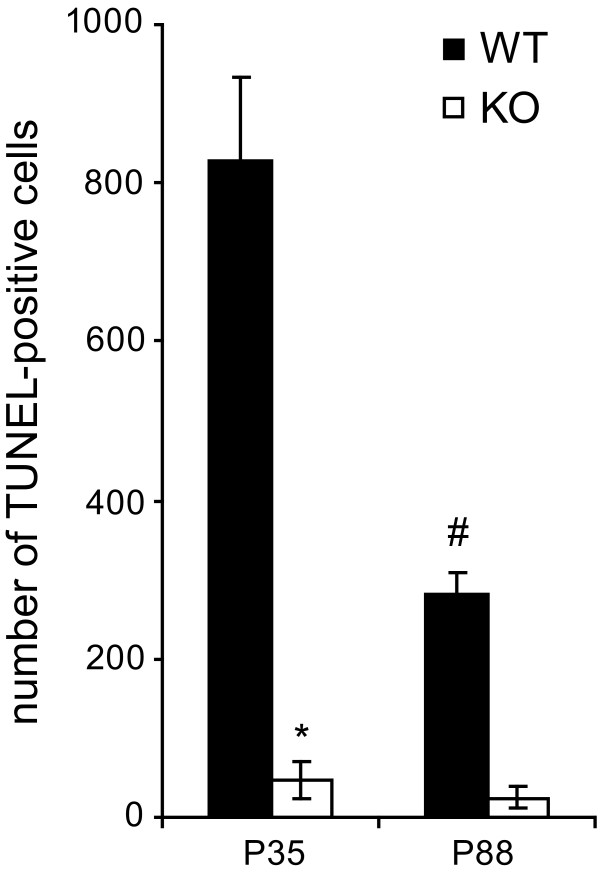
**Reduced cell death in the DG of cD2KO mice.** CD2KO mice have significantly less apoptotic cells in the DG than their WT litters (*p < 0.001). Numbers of TUNEL-positive cells decline with aging in WT mice (^#^*p* < 0.001) but remain stable in cD2KO animals.

TUNEL-positive, apoptotic cells were only rarely detected in the DG. A significantly lower number of apoptotic cells was observed in cD2KO compared to WT mice at P35 (WT: 830 ± 105, cD2KO: 48 ± 24; *p* < 0.001; Figure 8). This difference was still present at P88, albeit not statistically significant (WT: 282 ± 25, cD2KO: 24 ± 14; *p* = 0.1; Figure 8). A significant decline of TUNEL-positive nuclei was detected between P35 and P88 in WT animals (*p* < 0.001), but not in cD2KO mice. Independent of genotype or age, TUNEL-positive nuclei appeared preferentially in the SGZ.

## Discussion

Mice with targeted disruption of the cyclin D2 gene (cD2KO) have been reported to lack newborn neurons in the adult DG and olfactory bulb [27], an attribute making them a useful model to study the function of adult hippocampal neurogenesis. The temporal dependency of neurogenesis on cD2 during postnatal life has not been clearly evaluated. Hence, we systematically investigated the time course of neurogenesis in the DG of cD2KO mice by analyzing cD2KO and WT litters at seven time points during the first 10 months of life for their potential to generate new neurons in the DG. In brief, our results reveal that in cD2KO mice: 1) newborn cell numbers and hippocampal neurogenesis are significantly reduced, 2) neurogenesis virtually ceases at an age around P28, 3) morphology of the hippocampus is almost normal but it is smaller in size, 4) the GCL volume as well as DGC numbers are significantly reduced, 5) the lack of functional cD2 prevents the age-related increase in DGC numbers, and 6) apoptosis is substantially diminished compared to WT litters.

Adult neurogenesis persists throughout life, both in the SGZ of the hippocampus and in the SVZ/olfactory bulb, however, the potential to generate new neurons substantially declines with increasing age [28-34]. Using the exogenous marker BrdU and the endogenously expressed marker Ki67 to label and detect dividing cells, together with neuronal markers (NeuN and DCX), we confirmed the age-dependent change of adult neurogenesis in both, WT and cD2KO mice. Analyses of WT brains with Ki67 and BrdU revealed that the number of newborn cells declines by about 74% to 85%, respectively, between the ages of 3 to 9–10 months. This was paralleled by a decrease in the number of newborn neurons by 87%. The observed rates of age-related changes in cell proliferation are consistent with previous reports studying C57Bl/6 mice [29,35]. Differences in the percentages of Ki67- and BrdU-positive cell numbers in the present study may derive from the distinct characteristics of these markers [36,37] and the labeling protocol applied. Ki67 labels cells during all active phases of the cell cycle (G_1_, S, G_2_, and mitosis) and thus provides a snap shot of the proliferative situation at the time of sacrifice of the animal. The thymidine analogue BrdU is integrated into the DNA of cells during S-phase of cell cycle and retained in the progeny of dividing cells. It is to note, that the results obtained with the labeling scheme used in our study reflect a combination of proliferation and survival.

Noteworthy, we detected a substantial change in the absolute values of cell birth and neurogenesis due to the lack of functional cD2 at all time points analyzed. Whilst postnatal neurogenesis was still present in the DG of cD2KO mice, albeit at a lower level than in WT litters, adult-born neurons were rarely detectable. BrdU incorporation and neurogenesis were virtually absent as early as at P28. These data indicate that postnatal neurogenesis is controlled by cD2 together with at least one other D-type cyclin, and that the age at which DG neurogenesis becomes exclusively dependent on the expression of functional cD2 lies between P14 and P28.

Granule neurons of the DG are generated over a prolonged period starting early in embryogenesis (at E10 in mice; [38]) and continuing far into postnatal life [39,40]. During this period, germinative zones, containing the precursors of DGCs, arise in a consecutive manner with the primary dentate neuroepithelium lining the lateral ventricles arising first, giving rise to the adjacent secondary dentate matrix, which, around the time of birth, sends precursor cells towards the dentate anlage, forming the tertiary germinative matrix [39,41]. This proliferative zone constitutes the GCL of the DG from birth up to the third postnatal week. Significantly, between P20 and P30, proliferating cells become gradually confined to the SGZ, which serves as source of newly born neurons in the adult DG. This time window precisely correlates to the age at which DG neurogenesis virtually ceases in cD2KO mice.

Evidence suggests that developmental hippocampal neurogenesis takes place in the presence of at least one other D-type cyclin that probably compensates for cD2 deficiency in cD2KO mice. Supportingly, Glickstein et al. [42] demonstrated that cyclin D1 (cD1) and cD2 are expressed in a widely overlapping fashion in the germinative matrices that generate the DG. Moreover, they observed a successive change from cD1 towards cD2 expression in these germinative zones with ongoing developmental progression, suggesting a tendency of neuronal progenitors to become cD2 dependent during late-stage divisions. Thus, cD1 is most probably either functionally redundant to, or compensates for cD2 during development of cD2KO mice.

In addition, Glickstein and coworkers detected a small number of cD1 immunoreactive cells also in the adult SGZ of WT and cD2KO mice [42]. Moreover, these cells were proven to be proliferating by means of BrdU co-labeling. In the present study, we reaffirmed the existence of cD1 positive cells in the SGZ of adult WT and cD2KO mice ( Additional file 1). Most likely, cD1 expression in a subset of SGZ progenitors is responsible for the few residual newborn neurons found in the DG of cD2KO mice. In apparent contradiction to these results, only the cD2 transcript has been detected in neurospheres derived from the adult WT hippocampus [27]. However, the fact that neurospheres could be derived from the adult hippocampus of cD2KO mice [27], which furthermore expressed cD1 mRNA, strengthens the hypothesis that cD1 accounts for DG neurogenesis in cD2KO mice.

We observed no significant differences in neuronal differentiation when comparing the fraction of NeuN/BrdU double-labeled cells between WT and cD2KO mice. On average, 60% of the BrdU-labeled cells expressed NeuN after 28 days of differentiation, independent of genotype or age, which was in the range previously reported for mice and rats [30,43]. On the other hand, the fraction of actively dividing neuronal precursors (Ki67/DCX double-positive) was considerably reduced in cD2KO mice. In contrast to the BrdU/NeuN data – this result might be suggestive of differences in neuronal fate choice. However, this is unlikely since there is strong evidence that DCX-positive progenitors are already determined towards the neuronal lineage [44-46]. In the adult dentate gyrus, DCX is expressed by type 2b and type 3 progenitors, and by immature neurons [45]. Ki67 has been detected in both, type 2b and type 3 cells, denoting that these cells are proliferative [45]. The data of the present study indicate that cD2 is required for the division of at least a subset of DCX-positive progenitors. Whether these belong to the class of type 2b or type 3 transient amplifying cells remains to be determined. An alternative explanation that must be considered as reason for the apparently inconsistent data might be the very low number of dividing cells available for examination of neuronal fate choice in cD2KO mice, which could bias statistical testing.

However, in all groups of WT animals at different ages, ~25% of all Ki67-positive cells expressed DCX, indicating a stable rate of neuronal differentiation in young adults. These results fit well with previously reported observations in rats [47] and mice [29,45].

The number of granule neurons within the GCL was stereologically determined in mice aged P88 and P288. Estimations performed in WT mice were within the range reported in previous studies [29,31,48,49]. Similar as already shown for rats [50,51] and mice [49,52], we observed a significant increase in the number of DGCs between P88 and P288. In contrast, DGC numbers were significantly reduced (at P88 to 40% and at P288 to 35% of WT, respectively) and did not change with age in cD2KO mice. This indicates that though the lack of adult neurogenesis does not affect the number of neurons born earlier in life it prevents the age-related increase in DGC numbers. Thus, our results confirm previous reports suggesting that adult neurogenesis substantially adds new neurons to the hippocampal network rather than replacing existing neurons [49-51]. Further evaluation of the GCL revealed a significant volume reduction by ~49% in cD2KO mice at all ages examined. There were no age-dependent differences detectable within the groups of cD2KO and WT mice. Hence, while neurogenesis appears to be cumulative resulting in an increased number of DGCs over the lifetime of an animal, the volume of the DGL remains almost constant, irrespective of the presence or absence of adult neurogenesis. In agreement with previous studies [49,50], this indicates that the density of DGCs in the mouse increases with age.

As during development, apoptotic cell death seems to play an important role in the regulation of the final number of newborn neurons in the neurogenic zones of the adult brain [53]. In WT mice, we observed TUNEL-positive, apoptotic cells at low frequencies throughout the DG. These cells preferentially resided in the SGZ, with few TUNEL-positive cells also found in the GCL. Analysis of WT mice revealed that numbers of dying cells in the DG decreased with age (P35 vs. P88). This was consistent with previous reports describing a continuous decline in cell death from 2 months onwards in mice [29], or between 2 and 6 weeks in rats [54]. In contrast, in cD2KO mice the number of dying cells in the DG was strongly reduced (by >90%) and showed no age-related decline. Thus, the pattern and numbers of TUNEL-positive cells closely correlate to that of newborn neurons in the DG. Even if these numbers are not directly comparable, they might be useful to illustrate the relationship between rates of cell birth and death: One-month (P35) old WT mice exhibit the highest rate of cell birth and death, with both features decreasing with age (i.e. between P35 and P88). Mice lacking cD2 have significantly less apoptotic cells than WT mice corresponding to their lower rate of cell birth. While these mice show no age-dependent decline in adult neurogenesis from P28 onwards, the numbers of apoptotic cells also appear to remain stable. These findings support previous reports suggesting that adult hippocampal neurogenesis is counterbalanced by the simultaneous elimination of newborn neurons through apoptosis [53].

## Conclusions

The results of the present study emphasize the temporal dependency of hippocampal neurogenesis on cD2, and the importance of cD2 for adult neurogenesis. They suggest that postnatal neurogenesis is controlled by cD2 together with at least one other D-type cyclin. Hippocampal neurogenesis becomes increasingly dependent on cD2 during early postnatal development. Without functional cD2 it ceases at an age between P14 and P28, when the tertiary germinative matrix discontinues proliferative activity. These data indicate that cD2 becomes an essential requirement for ongoing neurogenesis with the transition from developmental to adult neurogenesis. Our data provide additional evidence that there is an ongoing, lifelong increase in the density of dentate granule cells due to adult neurogenesis.

Because of the lack of adult neurogenesis, cD2KO mice are a useful model to study the functional relevance of adult neurogenesis. In this context, our findings suggest that experimental interventions (such as physical activity, enriched environment, pharmacological treatments *etc.*) that interfere with hippocampal neurogenesis should not be started before neurogenesis becomes exclusively dependent on functional cD2.

## Methods

### Mice

All procedures involving living animals were carried out in strict compliance with the EC directive 86/609/EEC guidelines for animal experiments and were approved by the local government (Thueringer Landesamt, Bad Langensalza; permit no.: 02-012/07). Animals were housed under 12 h light/dark conditions with *ad libitum* access to food and water. The cyclin D2 gene was inactivated by excision of exons I and II [22]. Mice were kept as heterozygotes on C57Bl/6J background. Homozygous cyclin D2 knock out (cD2KO) and WT littermates (n ≥ 3 as indicated in Table 1) were used for all experiments.

### BrdU injection and tissue processing

Dividing cells were labeled by intraperitoneal injections of bromodeoxyuridine (BrdU, 50 mg/kg body weight; Sigma-Aldrich, St. Louis, MO, USA). Starting at either postnatal day (P) 7, P14, P28, P40, P60, P90, or P260, animals received BrdU every 8 h for 2 consecutive days (a total of 6 injections per animal; see Figure 1).

Twenty eight days thereafter, animals were deeply anesthetized and transcardially perfused with 4% paraformaldehyde in 0.1 M phosphate buffer, pH 7.4. The brains were removed and post-fixed in the same fixative for 24h at 4°C. Thereafter brains were cryoprotected in 30% sucrose (in 0.14 M PBS, 4°C), frozen in 2-methylbutan (−25 to −30°C) and stored at −80°C.

### Immunohistochemistry and -fluorescence

Coronal sections (40 μm) were treated for 30 min with 1.5% H_2_O_2_, blocked in TBS plus, containing 0.1% triton, 2% BSA and 3% donkey serum, and incubated over night at 4°C with primary antibodies: rat α-BrdU (1:500; AbD Serotec, Oxford, UK), rabbit α-Ki67 (1:400; Novocastra, Newcastle, UK), or rabbit α-cD1 (1:200; Thermo Fisher Scientific, Kalamazoo, MI, USA). Sections were then sequentially incubated in biotinylated secondary antibody (donkey α-rat or donkey α-rabbit, both 1:500; Dianova, Hamburg, Germany) for 3 h and Vectastain Elite ABC Kit (Vector Laboratories, Burlingame, CA, USA) for 1 h, followed by DAB (3,3`-Diaminobenzidine tetrahydrochloride hydrate; Sigma-Aldrich) signal detection. For BrdU immunohistochemistry, a denaturation step (30 min 2 N HCl) followed by 10 min neutralization in 0.1M borate buffer pH 8.5 was included after H_2_O_2_ treatment. For cD1 epitope retrieval, sections were steamed for 15 min in citrate buffer pH 6 before H_2_O_2_ treatment.

For immunofluorescence, a similar, but slightly modified protocol was applied: Briefly, to phenotype BrdU-positive cells, sections were pre-treated in TBS plus complemented with Fab α-mouse (1:20; Dianova) and then rinsed and incubated with primary antibodies: rat α-BrdU (1:500; AbD Serotec), mouse α-NeuN (1:500; Millipore/Chemicon, Billerica, MA, USA), and rabbit α-GFAP (1:1000; Synaptic Systems, Goettingen, Germany). To determine the phenotype of proliferating cells, sections were steamed for epitope retrieval (15 min in citrate buffer pH 6) and incubated with antibodies against Ki67 and doublecortin (goat α-DCX, 1:100; Santa Cruz Biotechnology, Santa Cruz, CA, USA). As secondary antibodies we used: donkey α-mouse Cy5, donkey α-rabbit RhX, donkey α-goat Cy5 (1:250; all from Dianova), and goat α-rat Alexa 488 (1:250; Molecular Probes/Invitrogen, Carlsbad, CA, USA). Nuclei were counterstained with DAPI.

### TUNEL

We used terminal deoxynucleotidyl transferase-mediated dUTP nick-end labeling (TUNEL) to detect nuclei with fragmented DNA, which is one of the hallmarks of late-state apoptosis. Every 24^th^ 40 μm-coronal section was rinsed in TBS and steamed for 15 min in 10 mM sodium citrate buffer (pH6). After cooling, sections were permeabilized in TBS/0.1% triton and incubated for 1h at 37°C with the TUNEL reaction mixture containing TdT and TMRred-dUTP (Roche, Mannheim, Germany), followed by DAPI counterstaining to visualize nuclear profiles.

### Volumetric analyses

Measurements were taken in every sixth 40 μm coronal section stained with cresyl violet acetate (Sigma-Aldrich). Sections were digitized at appropriate magnification and the areas of the brain, the hippocampus and the dentate granule cell layer (GCL) were measured using ImageJ software (NIH). Volumes (V) were calculated as V = ΣA · *i* · *d*, according to Cavalieri´s principle, with A representing the sum of areas from both hemispheres of each section, *i* the interval between the sections, and *d* the section thickness, respectively.

### Data quantification and statistical analysis

Total numbers of BrdU- and Ki67-positive cells were counted in every 6^th^ section throughout the subgranular and granular cell layers of the entire DG using a Zeiss Axioplan 2 microscope (Carl Zeiss AG, Oberkochen, Germany). The resulting numbers were multiplied by 6 to obtain an estimate of the total numbers of BrdU-positive cells in the complete DG. A similar approach was applied for quantification of TUNEL-positive nuclei numbers.

For phenotyping of BrdU-positive cells, random fields of DG containing BrdU-positive cells were selected in every 12^th^ section and z-stacks were scanned by confocal laser microscopy (LSM510, Zeiss). Phenotypes of 50–100 BrdU-positive cells per DG were determined in WT mice aged up to P90 and in cD2KO up to P14. BrdU-incorporation in the DG of cD2KO mice older than P14 and of P260 WT mice was sparse, hence the numbers of BrdU-positive cells that were phenotyped in these animals were less than 50. The percentage of co-labeled cells was calculated and absolute numbers were obtained by multiplying the percentage with the total numbers of BrdU-positive cells. A similar procedure was applied to study the co-localization of Ki67 and DCX.

Absolute numbers of DGCs were estimated stereologically (optical fractionator principle; StereoInvestigator, MBF Bioscience, Williston, USA; [55]) in a series of every 12^th^ DAPI-stained 40 μm-sections. For this purpose, a 70 x 70 μm grid was superimposed over each section and DGCs were counted in 10 x 10 μm counting frames using a 100x oil-immersion objective. Cells that were in sharp focus at the top and bottom (10%) focal planes were disregarded to avoid over-sampling and bias due to tissue preparation artifacts. Total DGC number (*N*) was calculated according to the equation *N* = Σ*Q*^-^ × (1/*ssf*) × (1/*asf*) × (1/*hsf*), with *Q*^*-*^ = number of counts, *ssf* = section sampling fraction, *asf* = area sampling fraction, and *hsf* = height sampling fraction (optical dissector height/average mounted section thickness). Calculation of the coefficient of error (CE) as estimator of accuracy of the probe runs was based on the Scheaffer method [56].

If not indicated otherwise, statistical comparisons were performed using 2-way ANOVA followed by Tukey test for multiple comparisons. In case the variables did not meet the assumptions for parametrical tests, data were *ln*-transformed before statistical testing. Data represent mean ± SEM, *p*-values < 0.05 were considered statistically significant.

## Competing interests

The authors declare that they have no competing interests.

## Authors’ contributions

AA performed immunohistochemistry, confocal analyses and cell countings and participated in data analysis and manuscript preparation. AU conceived and designed the experiments, carried out the TUNEL analysis and granule cell quantification, participated in data analysis and interpretation, and wrote the manuscript. OWW participated in study design and revised the manuscript. All authors read and approved the final manuscript.

## Supplementary Material

Additional file 1 Illustration of cD1 expression in the dentate gyrus of cD2KO and WT mice. CD1 positive cells were found scattered throughout the dentate gyrus of both, WT and cD2KO animals, with few cD1 positive cells located in the subgranular cell layer (arrowheads). The dashed line indicates the granule cell layer. Scale bar: 25 μm. Click here for file
