# Supplementary figures and images for: Age-dependent kinetics of dentate gyrus neurogenesis in the absence of cyclin D2
Source: BMC Neurosci. 2012 May 7;13:46. doi: 10.1186/1471-2202-13-46 (PMC3403990; doi:10.1186/1471-2202-13-46)

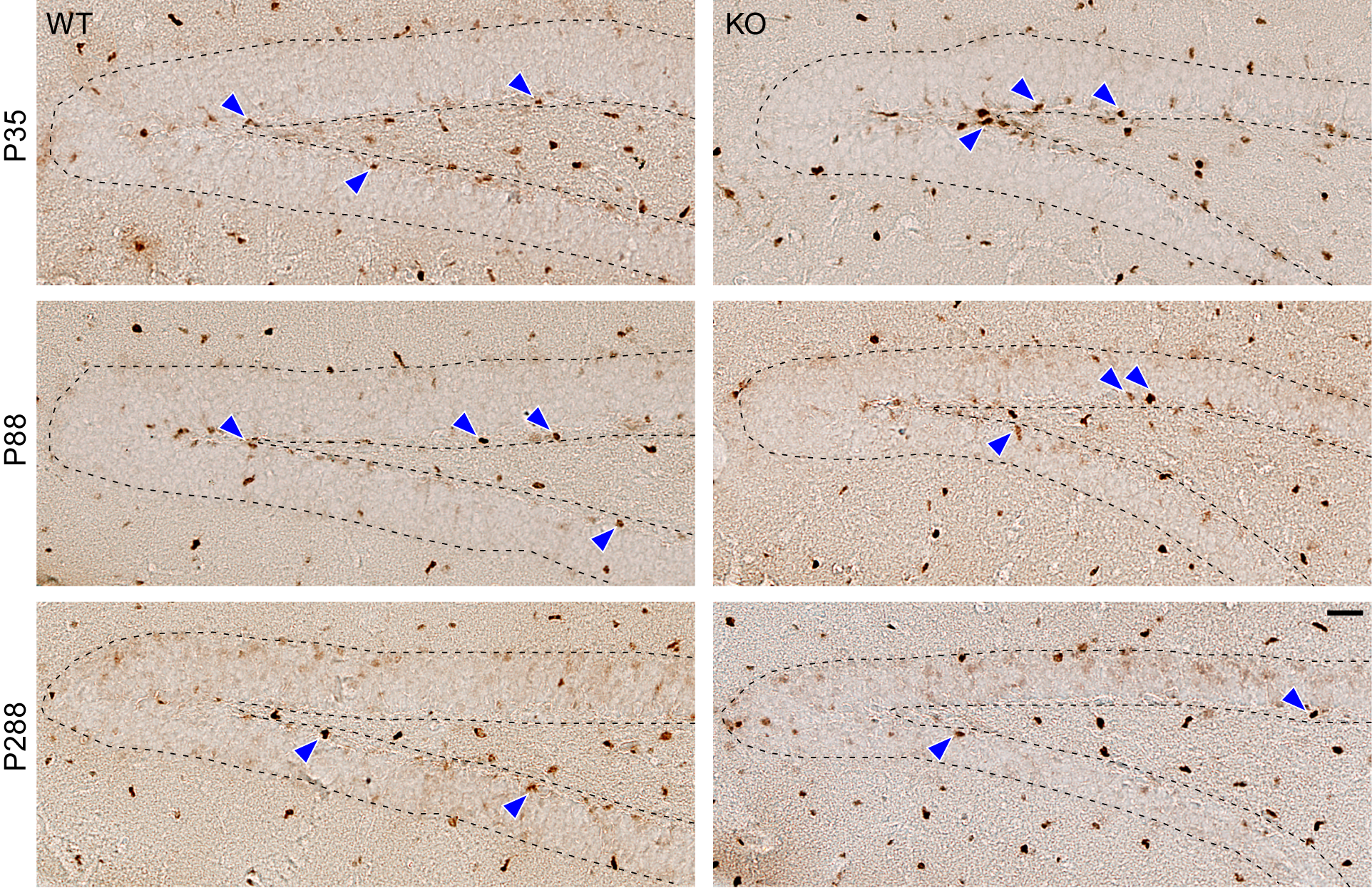

Supplement: Additional file 1 — Illustration of cD1 expression in the dentate gyrus of cD2KO and WT mice. CD1 positive cells were found scattered throughout the dentate gyrus of both, WT and cD2KO animals, with few cD1 positive cells located in the subgranular cell layer (arrowheads). The dashed line indicates the granule cell layer. Scale bar: 25 μm. [file 1471-2202-13-46-S1.tiff]
